# Generalizability of machine learning in predicting antimicrobial resistance in *E. coli*: a multi-country case study in Africa

**DOI:** 10.1186/s12864-024-10214-4

**Published:** 2024-03-18

**Authors:** Mike Nsubuga, Ronald Galiwango, Daudi Jjingo, Gerald Mboowa

**Affiliations:** 1 Department of Immunology and Molecular Biology, School of Biomedical Sciences, College of Health Sciences, Makerere University, P.O Box 7072, Kampala, Uganda; 2 Department of Computer Science, College of Computing and Information Sciences, Makerere University, P.O Box 7062, Kampala, Uganda; 3 The African Center of Excellence in Bioinformatics and Data-Intensive Sciences, Infectious Diseases Institute, College of Health Sciences, Makerere University, P.O Box 22418, Kampala, Uganda; 4 Africa Centres for Disease Control and Prevention, African Union Commission, P.O Box 3243, Roosevelt Street, Addis Ababa, W21 K19 Ethiopia; 5 Faculty of Health Sciences, University of Bristol, Bristol, BS40 5DU UK; 6 Jean Golding Institute, University of Bristol, Bristol, BS8 1UH UK

**Keywords:** Antimicrobial resistance, *E. coli*, Machine learning, Africa, Whole-genome sequencing

## Abstract

**Background:**

Antimicrobial resistance (AMR) remains a significant global health threat particularly impacting low- and middle-income countries (LMICs). These regions often grapple with limited healthcare resources and access to advanced diagnostic tools. Consequently, there is a pressing need for innovative approaches that can enhance AMR surveillance and management. Machine learning (ML) though underutilized in these settings, presents a promising avenue. This study leverages ML models trained on whole-genome sequencing data from England, where such data is more readily available, to predict AMR in *E*. *coli*, targeting key antibiotics such as ciprofloxacin, ampicillin, and cefotaxime. A crucial part of our work involved the validation of these models using an independent dataset from Africa, specifically from Uganda, Nigeria, and Tanzania, to ascertain their applicability and effectiveness in LMICs.

**Results:**

Model performance varied across antibiotics. The Support Vector Machine excelled in predicting ciprofloxacin resistance (87% accuracy, F1 Score: 0.57), Light Gradient Boosting Machine for cefotaxime (92% accuracy, F1 Score: 0.42), and Gradient Boosting for ampicillin (58% accuracy, F1 Score: 0.66). In validation with data from Africa, Logistic Regression showed high accuracy for ampicillin (94%, F1 Score: 0.97), while Random Forest and Light Gradient Boosting Machine were effective for ciprofloxacin (50% accuracy, F1 Score: 0.56) and cefotaxime (45% accuracy, F1 Score:0.54), respectively. Key mutations associated with AMR were identified for these antibiotics.

**Conclusion:**

As the threat of AMR continues to rise, the successful application of these models, particularly on genomic datasets from LMICs, signals a promising avenue for improving AMR prediction to support large AMR surveillance programs. This work thus not only expands our current understanding of the genetic underpinnings of AMR but also provides a robust methodological framework that can guide future research and applications in the fight against AMR.

**Supplementary Information:**

The online version contains supplementary material available at 10.1186/s12864-024-10214-4.

## Background

Antimicrobial resistance (AMR) is a pressing global health challenge that threatens human and animal well-being [1]. Recognized as a priority by the World Health Organization (WHO) and the United Nations General Assembly [2], AMR’s unchecked proliferation could lead to catastrophic consequences, with Africa alone projected to account for millions of annual deaths by 2050 [3]. In 2019, reports showed that AMR all-age death rates were highest in some low- and middle-income countries (LMICs), making AMR not only a major health problem globally but a particularly serious problem for some of the poorest countries in the world [4].

The WHO launched the Global Antimicrobial Resistance and Use Surveillance System (GLASS) to enhance AMR evidence base for priority pathogens including *Escherichia coli, Klebsiella pneumoniae, Acinetobacter baumannii, Staphylococcus aureus, Streptococcus pneumoniae, Salmonella spp *and others. While capacities for antimicrobial susceptibility testing (AST) exist across Africa, they are unevenly distributed and often limited in scope, particularly in LMICS. The COVID-19 pandemic has however catalyzed the broader adoption of Next-Generation Sequencing (NGS) platforms in Africa, now increasingly available to support a range of disease surveillance programs, including AMR. This technological advance offers a valuable complement to traditional AST methods, although the distribution and accessibility of NGS capabilities remain variable across the continent.

In Uganda, available data indicates concerning levels of drug resistance among *E*. *coli* strains (45.62%) with substantial resistance to key antibiotics [5]. Similarly, in Tanzania and Nigeria, studies have highlighted the growing challenge of AMR, reflecting patterns of resistance that may differ from other regions, thereby necessitating localized surveillance and tailored predictive models [6]. These countries exemplify the diverse AMR landscape across Africa and underscore the need for enhanced detection methods and strengthening diagnostic programs [7–9].

Overall, the increasing availability of whole-genome sequence (WGS) data in dedicated databases, exemplified by tools like CARD and Resfinder, has facilitated the identification of antibiotic resistance determinants [10, 11]. Existing approaches for detecting AMR from microbial whole-genome sequence data, such as rule-based models relying on identifying causal genes in databases, have high accuracy for some common pathogens but are limited in detecting resistance caused by unknown mechanisms in other major pathogenic strains. Machine learning techniques, including random forest, support vector machines, and neural networks have shown great promise in predicting antimicrobial resistance [12]. These methods excel in capturing complex patterns within large datasets and can directly learn valuable features from genomic sequence data without relying on assumptions about the underlying mechanisms of AMR. Previous studies using machine learning have demonstrated success in predicting AMR and pathogen invasiveness from genomic sequences [13–18]. Despite this potential, the application of machine learning for AMR prediction has not been widely explored in LMICs, often due to data scarcity and the underrepresentation of AMR genetic determinants within reference databases [19].

To bridge this gap, we adopted a cross-continental approach, training machine learning models on data from England and validating them on datasets from Uganda, Tanzania and Nigeria. This strategy aimed to evaluate the efficacy of machine learning in predicting AMR for *E. coli* and assess the models’ generalizability across diverse African settings and datasets. By leveraging microbial genomic data and advanced machine learning techniques, this study endeavored to enhance the accuracy and efficiency of AMR prediction, thus contributing significantly to the global battle against AMR. This comprehensive analysis provides crucial insights into the practical implementation and scalability of AMR prediction strategies, especially in LMICs where genomic data is limited and the burden of AMR is disproportionately high.

## Methods

### Study design

This was a cross-sectional study utilizing data collected in the past years to explore associations between predictors and outcomes.

### Sample size

In this study, two datasets, referred to as the Africa data and the England data of *E. coli* strains were used.

**Table 1:** Overview of the data

Antibiotic	CIP		CTX		AMP	
Source	Africa	England	Africa	England	Africa	England
Resistant	82	266	92	115	166	245
Susceptible	92	1228	16	1313	9	167
Total	174	1494	108	1428	175	412

### Data description

The study focused on three antibiotics ciprofloxacin (CIP), ampicillin (AMP) & cefotaxime (CTX). Each of these represented an antibiotic from a different class of antibiotics (penicillins, cephalosporins, and fluoroquinolones). They are broad-spectrum antibiotics with activity against numerous Gram-positive and Gram-negative bacteria, including *E. coli.* These drugs were selected based on their increasing prevalence of resistance as reported in the GLASS report [5]. In addition, data on resistance to these drugs was available in the study datasets, making them an ideal choice for the study. The study utilized data from one of the largest complete *E. coli* datasets that were already available online from the National Center for Biotechnology Information, eliminating the need for additional data collection efforts. We categorised the data into two primary datasets:

#### England dataset

Comprising of 1,496 samples for ciprofloxacin; 1,428 for cefotaxime; and 1,396 for ampicillin. The dataset was collected from England and consisted of WGS of 1509 *E. coli* strains and corresponding phenotypic information [20]. This data was collected in England by the British Society for Antimicrobial Chemotherapy and from the Cambridge University Hospitals NHS Foundation Trust as part of a longitudinal survey of *E. coli* to contextualize the ST131 lineage within a broader *E. coli* population. This data was made publicly available by the Wellcome Trust Sanger Institute (Accession: PRJEB4681).

#### Africa dataset

Comprising of data from Uganda, Tanzania and Nigeria. The first Africa dataset consisted of samples collected from pastoralist communities of Western Uganda to study phylogenomic changes, virulence, and resistant genes. It contained WGS data for a total of 42 *E. coli* strains [21]. These were isolated from stool samples from both humans (*n* = 30) and cattle (*n* = 12) collected between January 2018– March 2019. WGS was carried out at facilities of Kenya Medical Research Institute -Wellcome Trust, Kilifi. The data is made publicly available by the author in a repository (DOI 10.17605/OSF.IO/KPHRD). The second Africa dataset consisted of 73 samples collected from both Uganda (*n* = 40) and Tanzania (*n* = 33) in a study that was unravelling virulence determinants in extended-spectrum beta-lactamase-producing *E*. *coli* from East Africa using WGS [22]. The third dataset consisted 68 samples collected from Nigeria as part of a study looking at WGS data from *E*. *coli* isolates from South-West Nigeria hospitals [23] (Table 1).

The samples that had not been screened for AST were removed from the dataset.

### Variant calling of whole-genome sequencing data

The raw WGS paired-end reads were first quality checked and filtered by fastp 0.23.4 using its default parameters: adapter detection and trimming, sliding window quality filtering with a threshold of Q20, end trimming for low quality bases and removing reads shorter than 15 bp post-trimming [24]. The filtered reads were aligned to the *E. coli* K-12 substr. MG1655 U00096.3 complete genome using Burrows-Wheeler Aligner-mem (0.7.17-r1188) algorithm with default seed length of 19, bandwidth of 100, and off-diagonal X-dropoff of 100 [25]. BCFtools 1.18 was used for calling variants with a minimum of depth coverage of 10x and allelic frequency of 0.9 [26]. SAMtools 1.18 was used to sort the aligned reads and BCFtools 1.18 was used to filter the raw variants applying default filtering thresholds, including a minimum read depth of 2, SNP quality of 20 [27]. The entire bioinformatics workflow was subsequently executed on the Open Science Grid High Throughput Computing infrastructure [28, 29].

### SNPs pre-processing and encoding

**Fig. 1:**
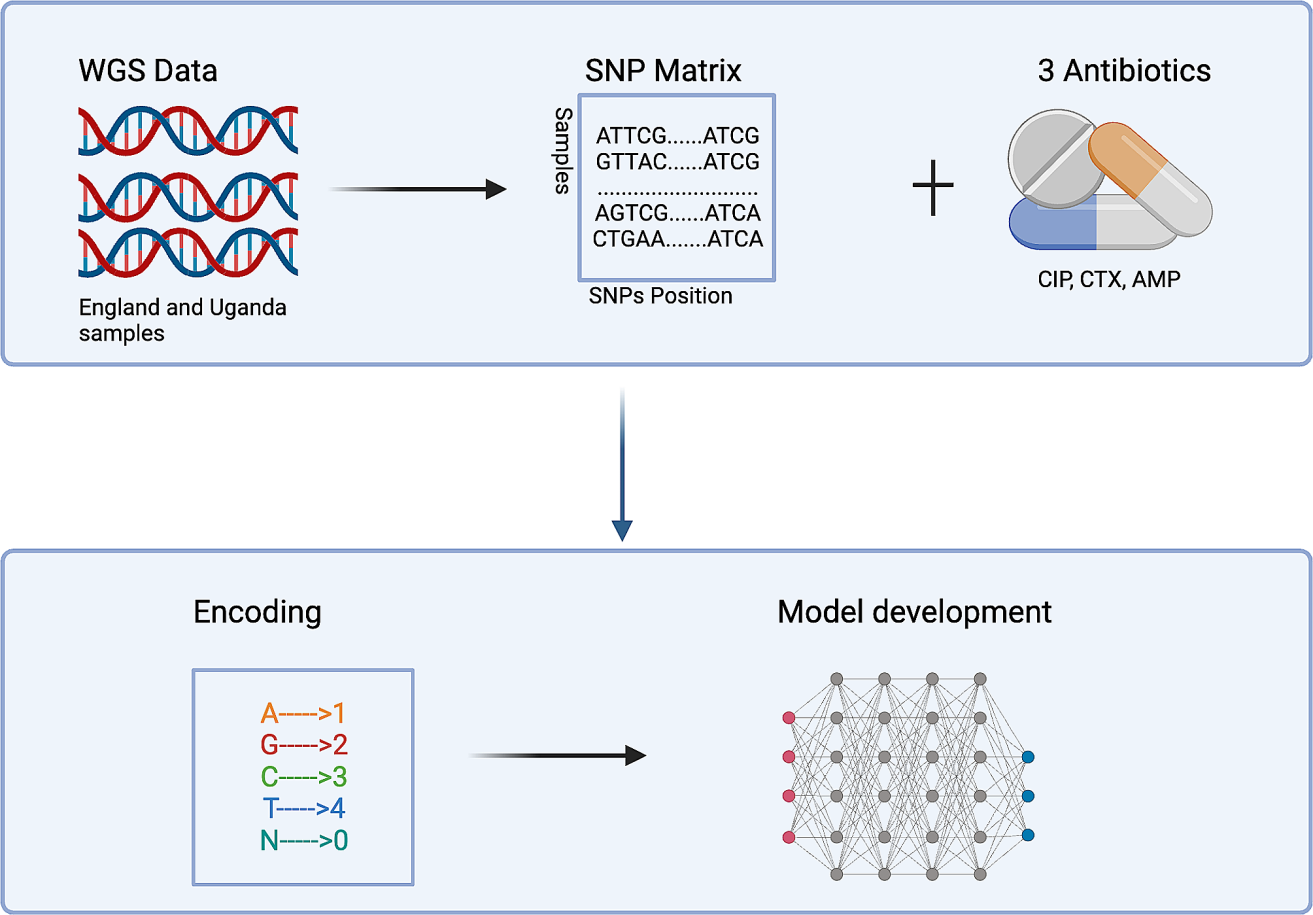
Illustration of the preprocessing and encoding process of the SNPs. Created with Biorender.com

We employed a previously established methodology for constructing the SNP matrix from the VCF files. First, the reference alleles, variant alleles, and their positions from the VCF files were extracted and merged with the isolates based on the position of the reference alleles. A SNP matrix was built where the rows represented the samples, and the columns represented the variant alleles [15]. The SNPs were converted from characters to numbers through categorical encoding where the categories are converted to numbers. The SNPs were encoded for machine learning using label encoding, where the A, C, G, T in the SNP matrix were converted to 1,2,3,4 (Fig. 1). It is acknowledged that certain machine learning models could misconstrue these as ordinal values; however based on previous studies demonstrating minimal performance difference between label, one-hot and Frequency Chaos Game Representation encoding methodologies [15], label encoding was selected for its computational efficiency in handling large genomic datasets. The missing values encoded as *N* were converted to 0. The gene positions that had more than 90% as null were removed and the remaining were selected for machine learning. The antibiotic phenotypes were encoded as binary values: ‘S’ for susceptible was mapped to 0, and ‘R’ for resistant was mapped to 1.

### Machine learning

We trained eight machine learning algorithms, each selected for its unique capabilities in predictive modeling. The training of these models was conducted individually for each antibiotic, focusing on one antibiotic at a time to ensure the specificity and accuracy of the predictions. Logistic Regression (LR) provided a baseline for binary classification, and Random Forest (RF) and Gradient Boosting (GB) were chosen for their effectiveness in handling high-dimensional data and intricate relationships. Support Vector Machines (SVM) were implemented with a sigmoid kernel, optimized through hyperparameter tuning to a *C* parameter of 9.795846277645586 and gamma set to ‘auto’. Feed-Forward Neural Networks (FFNNs), designed using Keras 2.12.0, consisted of an input layer with 64 neurons, a hidden layer with 32 neurons, and an output layer with one neuron, using binary cross-entropy loss and the Adam optimizer. The FFNN was trained for 20 epochs with a batch size of 32, with hyperparameter tuning improving its configuration. XGBoost (XGB) with xgboost 1.7.6, LightGBM (LGB) using lightgbm 4.1.0, and CatBoost using catboost 1.2.2 were implemented with default parameters, leveraging their efficiencies with large-scale data.

All models were implemented using Scikit-learn version 1.3.2, except for FFNNs which were implemented in Keras. Hyperparameter tuning was conducted for SVM and FFNNs using scikit-learn’s RandomizedSearchCV, which helped identify the most effective configurations for these models. The training was performed on both originally imbalanced and balanced datasets. For balancing, a simple random down-sampling approach was employed to reduce the majority class, enabling us to assess the impact of class distribution on model performance.

This comprehensive approach, involving diverse algorithms and hyperparameter tuning, allowed for an exhaustive evaluation of predictive models in the detection of AMR, under varied dataset conditions.

### Statistical evaluation

The machine learning models were optimized using five times 5-fold stratified cross-validation. For the final evaluation of the data from Africa, the performance was analyzed on the raw public dataset and on a balanced set using a downsampling strategy. The models were evaluated using the receiver operating characteristics curve (ROC) and the area under the curve (AUC). Precision, recall, f1-score, and accuracy for all models were calculated. In order to determine the statistical significance of the differences in AUC scores between models, we employed Tukey’s Honestly Significant Difference (HSD) test [30]. This test is appropriate for comparing all possible pairs of groups in a family of models without increasing the risk of Type I errors that multiple comparisons may induce. The significance threshold was set at α = 0.05, indicating that differences with p-values less than this threshold were considered statistically significant. The pairwise comparisons were conducted using statsmodels 0.14.0.

### Identification of genes

**Fig. 2:**
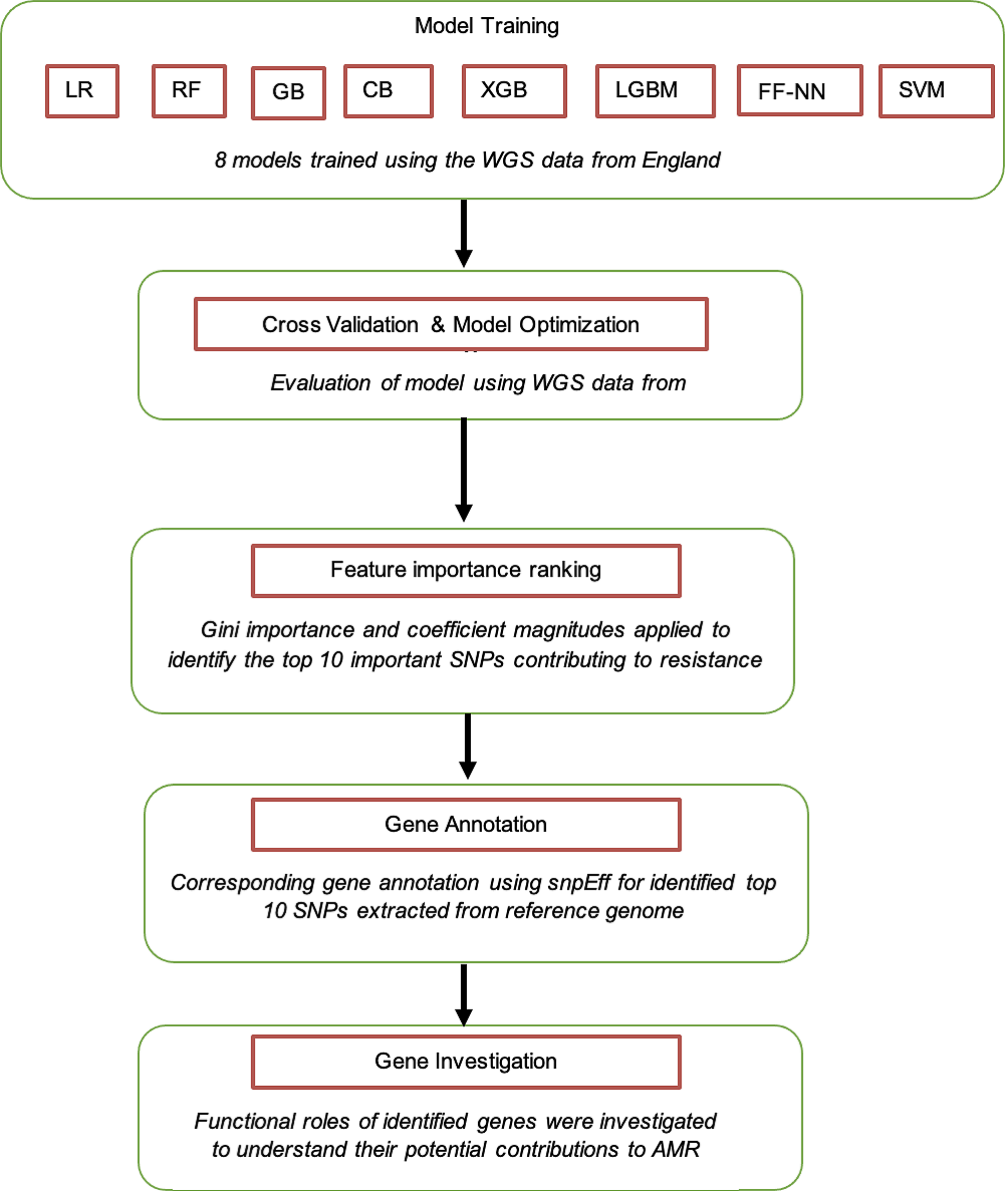
Flowchart showing how genes were identified

To identify the top 10 most important features for the models mentioned, the methods for calculating feature importance varied between models. For tree-based models like Random Forest, Gradient Boosting, XGBoost, LightGBM, and CatBoost, we utilized the feature_importances attribute, which quantifies the contribution of each feature to the model’s prediction. In Logistic Regression, feature importance was deduced from the absolute values of the coefficients. The SVM model employed the coefficients’ absolute values for linear kernels and SelectKBest with the chi2 method for non-linear kernels. For the Keras Neural Network model, we averaged the absolute values of the weights in the first layer, reflecting the relevance of each feature in the model. The corresponding gene annotations were extracted from the reference genome for the identified SNPs. By examining the functional roles of these genes, an investigation of their potential contribution to antibiotic resistance mechanisms in *E. coli* was done (Fig. 2).

## Results

### Performance of machine learning methods in predicting AMR

We assessed the performance of eight machine learning algorithms, including LR, RF, SVM, GB, XGB, LGB, CatBoost, and FFNN, in predicting antibiotic resistance in *E.* *coli.* Multiple metrics, such as accuracy, precision, recall, F1 score, and the area under the receiver operating characteristics (ROC) curve, were used for evaluation (Table 2). The models were optimized using 5-fold stratified cross-validation and confidence intervals recorded (Supplementary Material 1). Tukey’s Honestly Significant Difference (HSD) test was employed for pairwise comparisons of AUC scores.

**Table 2:** Performance of different machine learning methods for predicting AMR on England data

Antibiotic	Model	Accuracy	Precision	Recall	f1_score	auc_roc
CIP	Logistic Regression	0.85	0.60	0.48	0.54	0.83
	Random Forest	0.84	0.58	0.35	0.44	0.74
	SVM	0.87	0.68	0.50	**0.57**	**0.86**
	Gradient Boosting	0.86	0.72	0.33	0.46	0.83
	XGBoost	0.85	0.63	0.41	0.49	0.82
	LightGBM	0.85	0.61	0.43	0.50	0.84
	CatBoost	**0.87**	**0.81**	0.39	0.52	0.84
	Feed-Forward NN (Keras)	0.83	0.52	**0.50**	0.51	0.77
AMP	Logistic Regression	0.48	0.56	0.67	0.57	0.49
	Random Forest	0.43	0.52	0.57	0.54	0.44
	SVM	0.51	0.56	0.71	0.63	0.47
	Gradient Boosting	**0.58**	**0.63**	0.69	0.66	**0.52**
	XGBoost	0.53	0.61	0.57	0.59	0.51
	LightGBM	0.53	0.60	0.61	0.61	0.52
	CatBoost	0.51	0.56	**0.71**	0.63	0.48
	Feed-Forward NN (Keras)	0.52	0.58	0.67	0.62	0.47
CTX	Logistic Regression	0.91	0.47	0.38	0.42	0.77
	Random Forest	0.91	0.47	0.29	0.36	0.73
	SVM	**0.92**	**1.00**	0.04	0.08	0.79
	Gradient Boosting	0.91	0.33	0.12	0.18	**0.82**
	XGBoost	0.91	0.47	0.29	0.36	0.81
	LightGBM	**0.92**	0.57	0.33	0.42	0.81
	CatBoost	0.91	0.33	0.12	0.18	0.80
	Feed-Forward NN (Keras)	0.91	0.42	0.21	0.28	0.80

For CIP, we evaluated the models’ effectiveness considering the class imbalance issue. We applied a random down-sampling strategy but didn’t observe significant improvements. The FFNN emerged as the top performer with the highest mean AUC score (0.83), while SVM achieved the highest accuracy (0.87). HSD tests revealed significant performance differences between several pairs of models, specifically RF (*p* < 0.001) when compared to all the models.

For AMP, the SVM achieved the highest mean AUC score (0.72). GB had the highest F1 score and precision, and CB and SVM had the highest recall scores.

**Fig. 3:**
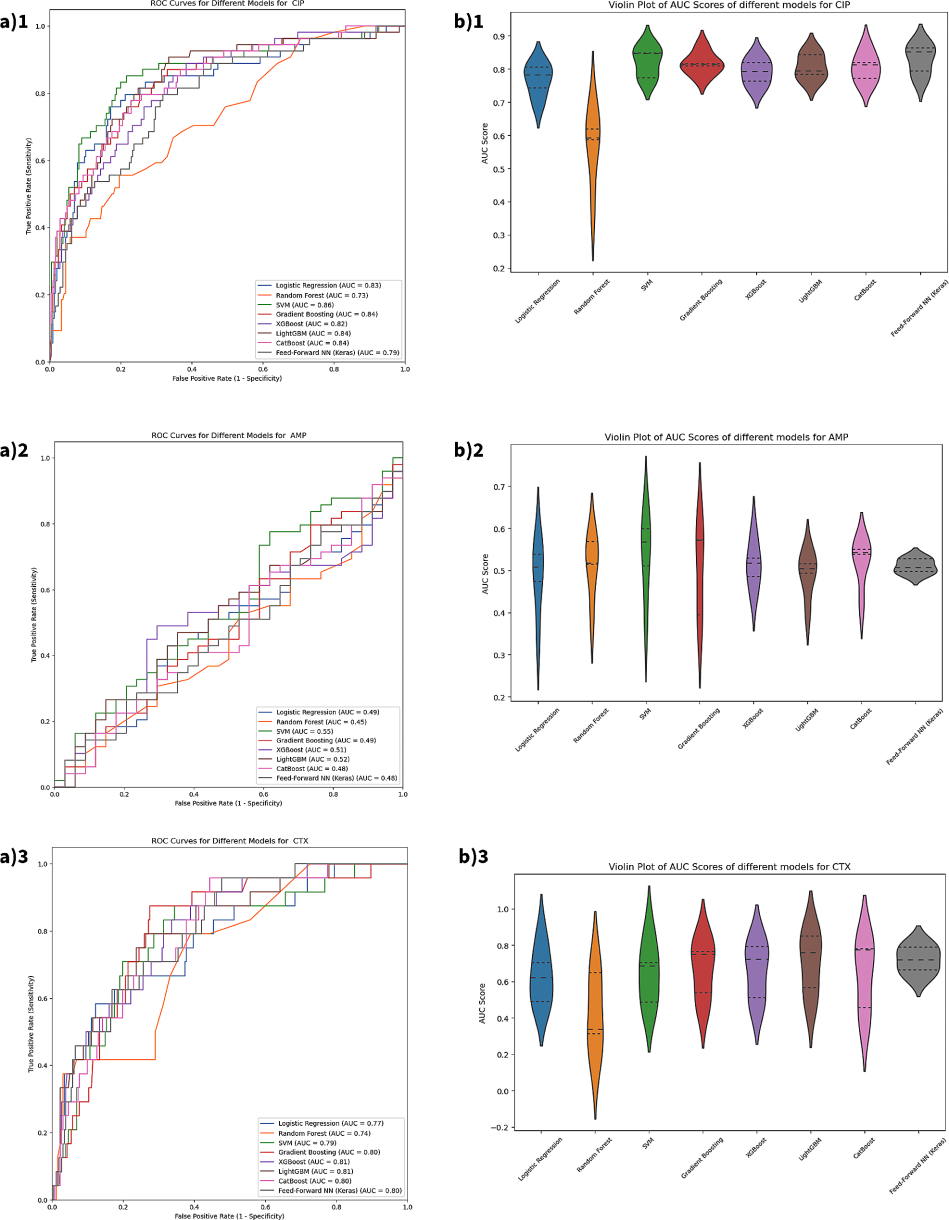
Performance of different machine learning methods for predicting AMR on England microbial sequence data

On the CTX, FFNN stood out with the highest mean AUC score (0.72), while SVM recorded the highest accuracy (0.92). The Random Forest model excelled in precision, and Logistic Regression had the highest F1 score (0.42) (Fig. 3).

### Evaluation of the machine learning models on the Africa data

We assessed the generalizability of our machine learning models on an external dataset from Uganda, Nigeria and Tanzania, consisting of up to 170 samples with a severe class imbalance issue. Performance metrics for each model on this dataset (Table 3).

**Table 3:** Performance of different machine-learning methods for predicting AMR on Africa data

Antibiotic	Model	Accuracy	Precision	Recall	f1_score	auc_roc
CIP	Logistic Regression	0.55	0.59	0.16	0.25	0.48
	Random Forest	0.50	0.48	0.68	0.56	0.53
	SVM	0.50	0.31	0.05	0.08	0.38
	Gradient Boosting	0.52	0.49	0.32	0.39	0.49
	XGBoost	0.57	0.67	0.17	0.27	0.56
	LightGBM	0.55	0.62	0.12	0.20	0.51
	CatBoost	0.56	0.61	0.17	0.27	0.58
	Feed-Forward NN (Keras)	0.56	0.71	0.12	0.21	0.49
AMP	Logistic Regression	**0.94**	0.95	0.99	**0.97**	0.60
	Random Forest	0.38	0.93	0.38	0.54	0.34
	SVM	0.86	0.94	**0.91**	**0.93**	0.57
	Gradient Boosting	0.59	0.96	0.59	0.73	**0.68**
	XGBoost	0.82	0.96	0.84	0.90	0.62
	LightGBM	0.34	0.89	0.35	0.50	0.38
	CatBoost	0.39	**0.98**	0.36	0.53	0.64
	Feed-Forward NN (Keras)	0.66	0.95	0.67	0.79	0.62
CTX	Logistic Regression	0.39	0.96	0.29	0.45	0.70
	Random Forest	0.20	0.88	0.08	0.14	0.61
	SVM	0.25	**1.00**	0.12	0.21	0.68
	Gradient Boosting	0.22	**1.00**	0.09	0.16	0.57
	XGBoost	0.44	0.94	0.36	0.52	0.65
	LightGBM	0.45	0.95	0.38	0.54	0.63
	CatBoost	0.24	0.86	0.13	0.23	0.55
	Feed-Forward NN (Keras)	0.15	0.00	0.00	0.00	0.63

In the external validation with the African dataset, the class imbalance presented varied challenges across different antibiotics. For CIP, the Logistic Regression model exhibited an accuracy of 0.55 and precision of 0.59, but a recall of only 0.16. The RF model achieved an accuracy of 0.50 and an AUC-ROC score of 0.53. SVM displayed an accuracy of 0.50, while GB showed an accuracy of 0.52 and a recall of 0.32. XGB had an accuracy of 0.57, and both LGB and CatBoost had accuracies just above 0.55, with CB also attaining an AUC-ROC of 0.58. The FFNN model did not identify any true positives.

For AMP, LR achieved an accuracy of 0.94 and a near-perfect recall. RF had a precision of 0.93 but a lower accuracy of 0.38. SVM’s performance was close to that of LR, with high accuracy and recall but a slightly lower AUC-ROC score of 0.57. GB, XGB, LGB, and CatBoost demonstrated solid accuracy and precision, albeit with varying AUC-ROC scores. The FFNN model’s accuracy was at 0.05.

**Fig. 4:**
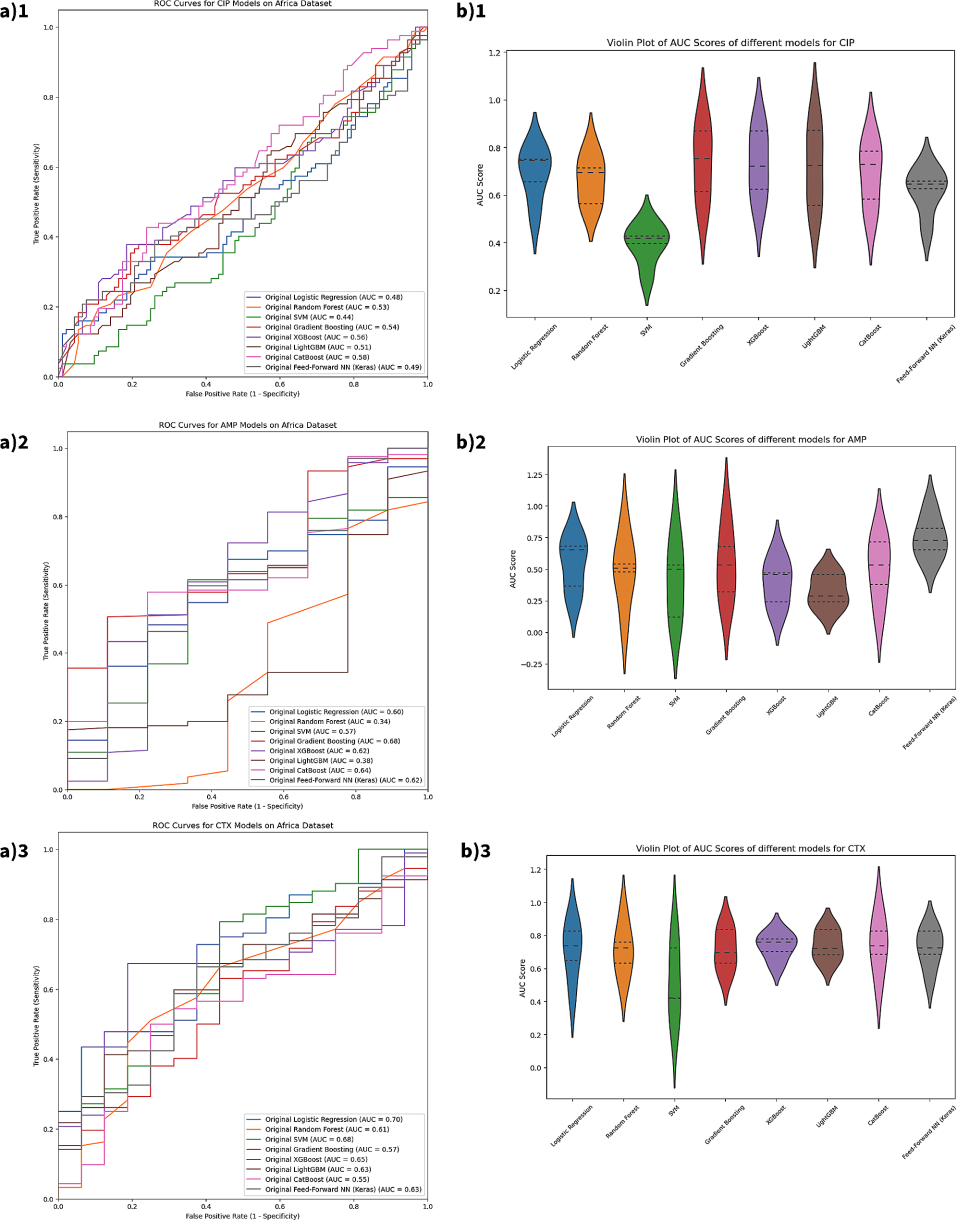
Performance of different machine learning methods for predicting AMR on Africa microbial sequence data

Regarding CTX, LR recorded an AUC-ROC of 0.39, RF exhibited high precision but low recall, and SVM had a precision of 1. GB had the highest accuracy among the models at 0.22 and the highest AUC-ROC score of 0.57. XGB and LGB showed higher accuracy and recall rates, with LGB achieving the highest recall of 0.38. The FFNN model again showed zero capacity for true positive identification (Fig. 4).

### Marker genes associated with antibiotic resistance

A crucial part of machine learning in the genomic field is to interpret the model’s results. In our case, the analysis of feature importance and interactions provided insights into which genetic mutations are most influential in predicting antibiotic resistance. For each model, we identified the top 10 features (SNP positions) with the highest importance scores, which reflect their contribution to the accuracy of the model’s predictions.

For instance, in the Logistic Regression model on CIP, the mutation at position ‘3589009’ has the highest importance score, followed by ‘4040529’, ‘1473047’, and so on. These positions potentially have a substantial impact on antibiotic resistance, as mutations in these areas of the gene could probably cause the bacteria to become resistant to specific antibiotics. The exact biological mechanism for this can be complex, involving changes in the gene’s protein product that might render an antibiotic ineffective (Table 4).

The models used different ways to calculate these importance scores, which is why they differ between models. Still, positions that are consistently high across different models can be a strong indicator of their significance in conferring antibiotic resistance.

**Table 4:** A table showing the top 10 mutation positions that have the most significant impact on the algorithms across all drags

	Logistic Regression	Random Forest	Gradient Boosting	XGBoost	LightGBM	CatBoost	Feed-Forward NN	SVM
	(‘Feature’, ‘Score’)	(‘Feature’, ‘Score’)	(‘Feature’, ‘Score’)	(‘Feature’, ‘Score’)	(‘Feature’, ‘Score’)	(‘Feature’, ‘Score’)	(‘Feature’, ‘Score’)	(‘Feature’, ‘Score’)
1	(‘3,589,009’, 0.47)	(‘1,008,149’, 0.002)	(‘2,189,699’, 0.05)	(‘4,374,394’, 0.04)	(‘4,040,529’, 27)	(‘4,428,325’, 2.39)	(‘4,614,839’, 0.03)	(‘1,008,149’, 151.24)
2	(‘4,040,529’, 0.39)	(‘3,284,134’, 0.004)	(‘3,230,190’, 0.05)	(‘1,008,149’, 0.03)	(‘4,437,351’, 27)	(‘3,230,190’, 1.5)	(‘4,428,325’, 0.02)	(‘3,009,381’, 131.13)
3	(‘1,473,047’, 0.34)	(‘4,240,504’, 0.002)	(‘1,008,149’, 0.05)	(‘3,284,134’, 0.02)	(‘2,160,560’, 26)	(‘2,189,699’, 1.38)	(‘4,615,406’, 0.02)	(‘4,172,848’, 119.69)
4	(‘3,088,751’, 0.32)	(‘1,263,246’, 0.003)	(‘3,284,134’, 0.04)	(‘3,615,876’, 0.02)	(‘3,680,451’, 25)	(‘1,008,149’, 1.26)	(‘4,437,351’, 0.02)	(‘4,445,374’, 108.66)
5	(‘3,117,412’, 0.31)	(‘4,453,756’, 0.002)	(‘1,263,246’, 0.03)	(‘2,189,699’, 0.02)	(‘3,118,273’, 21)	(‘3,284,134’, 1.08)	(‘4,458,693’, 0.02)	(‘3,418,178’, 107.1)
6	(‘3,118,273’, 0.31)	(‘2,824,686’, 0.002)	(‘4,240,504’, 0.03)	(‘1,239,691’, 0.01)	(‘4,428,325’, 21)	(‘4,615,406’, 1.08)	(‘4,452,347’, 0.02)	(‘2,791,632’, 106.19)
7	(‘1,324,783’, 0.3)	(‘4,374,394’, 0.003)	(‘4,374,394’, 0.03)	(‘3,230,190’, 0.01)	(‘4,481,654’, 21)	(‘3,584,376’, 0.99)	(‘3,200,153’, 0.02)	(‘1,264,526’, 104.35)
8	(‘1,720,453’, 0.28)	(‘4,286,100’, 0.002)	(‘3,373,941’, 0.03)	(‘2,792,739’, 0.01)	(‘2,480,806’, 20)	(‘4,445,374’, 0.97)	(‘4,443,644’, 0.02)	(‘4,172,776’, 103.49)
9	(‘4,446,973’, 0.28)	(‘3,614,716’, 0.002)	(‘3,615,876’, 0.02)	(‘3,021,822’, 0.01)	(‘3,589,009’, 18)	(‘3,681,794’, 0.96)	(‘4,434,509’, 0.02)	(‘4,218,643’, 102.63)
10	(‘2,778,469’, 0.28)	(‘1,466,504’, 0.002)	(‘3,681,794’, 0.02)	(‘3,373,941’, 0.01)	(‘2,035,257’, 16)	(‘4,269,331’, 0.96)	(‘3,584,376’, 0.02)	(‘3,300,216’, 100.93)

### Gene annotation

The identification of genetic SNPs associated with antibiotic resistance can shed light on the underlying genetic mechanisms that contribute to drug resistance in *E. coli* (Table 5). By analyzing the top SNPs from each predictive model, key marker genes that potentially play a role in antibiotic resistance were identified. For CIP, SNPs were identified in the following genes: *rlmL, yehB, rrfA, vciQ*, and *ygjK*. For AMP, the implicated genes include *rcsD, yjfI, tdcE, ugpB, ugpQ*, and *ggt*. Lastly, for CTX, SNPs were found in *ydbA, mltB, lomR, mppA, recD*, and *glyS*. The identified SNPs in these genes underscore the complex and multifactorial nature of antibiotic resistance in *E. coli*. A variety of biological processes, such as membrane transport, rRNA methylation, DNA repair, and cell wall synthesis, are potentially collectively implicated in the development of resistance. Further experimental validation of these marker genes is warranted to confirm their role in antibiotic resistance.

**Table 5:** A table showing the highest-scoring SNP positions and their corresponding genes. *SNP annotation is the functional implication and SNP impact is a classification of how a SNP might affect the function of the gene*

SNP Position	Gene location	Gene	SNP Annotation	SNP impact	Gene BioType	Drug
1,008,149	1,007,844–1,009,952	*rlmL*	Synonymous	LOW	Protein coding	CIP
2,189,699	2,188,430–2,190,910	*yehB*	Synonymous	LOW	Protein coding	CIP
4,040,529	4,040,517–4,040,636	*rrfA*	Intragenic	MODIFIER	Protein coding	CIP
1,324,783	1,324,746–1,326,641	*yciQ*	Missense	MODERATE	Protein coding	CIP
3,230,190	3,228,888–3,231,239	*ygjk*	Missense	MODERATE	Protein coding	CIP
2,313,886	2,313,488–2,316,160	*rcsD*	Synonymous	LOW	Protein coding	AMP
3,260,865	3,260,124–3,262,418	*tdcE*	Synonymous	LOW	Protein coding	AMP
3,591,222	3,591,009–3,592,325	*ugpB*	Synonymous	LOW	Protein coding	AMP
4,410,212	4,410,133–4,410,534	*yjfI*	Missense	MODERATE	Protein coding	AMP
2,824,686	2,824,491–2,825,576	*mltB*	Synonymous	LOW	Protein coding	CTX
1,427,523	1,427,389–1,427,598	*lomR*	synonymous	LOW	Protein coding	CTX
1,393,610	1,393,227–1,394,840	*mppA*	Missense	MODERATE	Protein coding	CTX
3,723,696	3,722,328–3,724,397	*glyS*	Synonymous	LOW	Protein coding	CTX

## Discussion

This study embarked on an explorative journey to understand the generalizability of machine learning models in predicting AMR in *E*. *coli*, utilizing datasets from England and multiple African countries. While the models showed promise on the England dataset, the application to the highly imbalanced African dataset illuminated significant challenges. The validation of machine learning models on the African dataset, which had a higher incidence of resistant strains compared to the training data from England, highlighted the challenges and potential of such tools; discrepancies in class distribution impacted performance measures like recall and precision, yet the robustness and real-world applicability of these models were affirmed when they successfully predicted resistance across varied datasets.

In the England dataset, models like SVM (Accuracy: 0.87, AUC-ROC: 0.86) and Logistic Regression (AUC-ROC: 0.77) demonstrated effectiveness. However, the transition to the African dataset, characterized by significant class imbalance, presented a stark contrast. For example, the Random Forest model experienced a decline in accuracy from 0.75 for CIP in the England dataset to 0.50 in the African dataset.

The performance of the models on the African dataset, particularly in terms of recall, highlights potential overfitting to the England dataset and the need for more generalizable models. The disparity in class distribution between the datasets—where the England dataset had a higher proportion of susceptible strains and the African dataset had a higher proportion of resistant strains—presented both challenges and opportunities.

A notable observation in this study is the impressive performance of the models for predicting ampicillin (AMP) resistance in the African dataset, despite their moderate performance on the England dataset. For AMP, models demonstrated substantial accuracy and recall in the African dataset (e.g., Logistic Regression: Accuracy 0.94, Recall 0.99, F1 0.97), highlighting their effectiveness in identifying true resistance cases. This success may be attributed to the distinct resistance mechanisms of AMP, which were perhaps better captured in the training data, leading to more accurate predictions in the validation dataset, or the data representation of the AMP training dataset which might have contained patterns that were more representative of the resistance seen in the African dataset.

Moreover, the process of down-sampling the England dataset for training, while fostering a balanced environment, did not uniformly enhance model performance. While down-sampled models showed a slight improvement for AMP, indicating that down-sampling might enhance the model’s sensitivity to specific resistance patterns associated with AMP, this effect was not as pronounced for CIP and CTX.

The identification of SNPs associated with antibiotic resistance can illuminate the genetic mechanisms driving drug resistance in *E. coli*. By analyzing the top SNPs from each predictive model, we identified key marker genes potentially involved in antibiotic resistance. For CIP, SNPs were identified in the following genes: *ugpC, rlmL, yciQ, ygjK, yehB, rrfA, ytfB*, and *yjjW*. These genes encode for various bacterial functions. For instance, *ugpC* is part of the glycerol-3-phosphate (G3P) transport system implicated in phospholipid biosynthesis, and RlmL is an enzyme involved in the methylation of ribosomal RNA (rRNA). *mdtC* is a component of multidrug efflux pump systems that can contribute to antibiotic resistance by actively pumping out antibiotics from bacterial cells. It’s important to note that while machine learning can highlight these genes as candidates, experimental validation is essential to confirm their roles in antibiotic resistance.

### Implications and applications

While our research concentrated primarily on three specific antibiotics, the methodology we’ve developed is versatile and readily adaptable for investigating other antibiotics and can be extended to resistance-associated SNPs in a variety of pathogens beyond just bacteria. This flexibility allows for a broader scope of study, opening the door for a comprehensive understanding of AMR mechanisms. In addition, the applicability of our approach extends beyond the realm of infectious diseases, holding promise for other branches of biomedical research, such as predicting resistance to cancer treatments by enabling precise targeted therapy.

### Limitations

While this study has provided valuable insights into predicting genotypic resistance to ciprofloxacin, ampicillin, and cefotaxime in *E. coli* strains, it is important to acknowledge several limitations that should be considered when interpreting the results. First, it is important to acknowledge the inherent limitation of focusing exclusively on SNPs as the single specific genomic factor. Antimicrobial resistance is a complex phenomenon influenced by various genomic drivers including resistance genes, insertion sequences, plasmids and AMR gene cassettes which collectively contribute to the intricate landscape of resistance mechanisms. Our study, by concentrating on SNPs, represents a deliberate simplification to ensure depth and clarity in our ML analysis, driven by data quality and the need for clinically interpretable models. However, we recognise that the exclusive emphasis on SNPs may not capture the entirety of the multifaceted interplay within resistance determinants.

Furthermore, it is worth noting that the validation of the models on Africa data presented some challenges. The availability of whole-genome sequence data from Africa was limited, resulting in a relatively small dataset for model evaluation. Additionally, the African dataset exhibited high-class imbalance, where certain resistance classes were significantly underrepresented. This imbalance can introduce bias and affect the performance metrics of the models. Due to our study’s uniqueness, traditional benchmarking might not capture our nuanced challenges. Future studies should explore alternative methodologies for a comprehensive evaluation of predictive models in diverse contexts.

Moreover, it is important to highlight that the performance of the models in this study is specific to the context of the datasets used, which may not fully represent the diversity and complexity of AMR patterns observed in other regions or populations. Therefore, caution should be exercised when generalizing the findings to different settings. Despite these limitations, this study provides a valuable foundation for future research and highlights areas for improvement and expansion. Incorporating additional variables, addressing the class imbalance, and expanding the dataset to include a more diverse range of sequences would enhance the robustness and applicability of the models. Overall, while the findings of this study contribute to our understanding of genotypic resistance prediction, it is important to recognize these limitations and consider them in the broader context of AMR research.

## Conclusion

In conclusion, our study highlights the complex interplay between data composition, model training approaches, and predictive accuracy in the context of AMR. The impressive performance of models for AMP in the African dataset despite their moderate performance in the England dataset underscores the potential of machine learning in AMR prediction, given appropriate training and validation strategies.

The findings from this study serve as a crucial reminder of the complexities involved in applying machine learning models to predict AMR across diverse settings. It emphasizes the importance of developing robust, adaptable, and generalizable machine learning tools, capable of handling varied data landscapes and resistance mechanisms. Future research should focus on integrating larger and more diverse datasets while exploring innovative methods to maintain a balance between dataset size and class distribution, thus advancing the development of machine learning tools in the global fight against antimicrobial resistance.

As the threat of antimicrobial resistance continues to rise, the successful application of these models - particularly on the African dataset, signals a promising avenue for improving AMR detection and treatment strategies. This work thus not only expands our current understanding of the genetic underpinnings of antibiotic resistance but also provides a robust methodological framework that can guide future research and applications in the fight against antimicrobial resistance.

### Electronic supplementary material

Below is the link to the electronic supplementary material.

Supplementary Material 1

## Data Availability

The source code in data preparation and model training is provided on the GitHub page: https://github.com/KingMike100/mlamr.

## Data availability

The data that supports the findings of this study is publicly available at EBI from https://www.ebi.ac.uk/ena/browser/view/PRJEB4681 and https://osf.io/kphrd/.

## Abbreviations

AMP: Ampicillin
AMR: Antimicrobial Resistance
AST: Antimicrobial Susceptibility Testing
CIP: Ciprofloxacin
CNN: Convolutional Neural Network
CRyPTIC: Comprehensive Resistance Prediction for Tuberculosis:an International Consortium
CTX: Cefotaxime
DNA: Deoxyribonucleic Acid
GLASS: World health Organization Global Antimicrobial Resistance and Use Surveillance system
LR: Logistic Regression
ML: Machine Learning
NCBI: National Centre for Biotechnology Information
PCR: Polymerase Chain Reaction (PCR)
qPCR: Real-time Polymerase Chain Reaction
REC: Research Ethics Committee
RF: Random Forest
RNA: Ribonucleic acid
SVM: Support Vector Machine
UNCST: Uganda National Council for Science and Technology
WGS: Whole Genome Sequencing
WHO: World Health Organization
