# Supplementary material for: Generalizability of machine learning in predicting antimicrobial resistance in E. coli: a multi-country case study in Africa
Source: BMC Genomics. 2024 Mar 18;25:287. doi: 10.1186/s12864-024-10214-4 (PMC10946178; doi:10.1186/s12864-024-10214-4)
Supplement: Supplementary file 1 — Supplementary Material 1 [file 12864_2024_10214_MOESM1_ESM.docx]

**Supplementary information for Nsubuga et al**

**Supplementary Material 1**

|  | AUC Mean | AUC Std Dev | AUC 95% CI Lower | AUC 95% CI Upper |
| --- | --- | --- | --- | --- |
| Logistic Regression | 0.76722766 | 0.04397415 | 0.72868261 | 0.8057727 |
| Random Forest | 0.56725898 | 0.0960713 | 0.48304877 | 0.65146919 |
| SVM | 0.82186516 | 0.04002479 | 0.78678189 | 0.85694843 |
| Gradient Boosting | 0.81051004 | 0.03073834 | 0.78356669 | 0.83745338 |
| XGBoost | 0.79064686 | 0.03538657 | 0.75962916 | 0.82166456 |
| LightGBM | 0.80726596 | 0.03472556 | 0.77682766 | 0.83770426 |
| CatBoost | 0.80474038 | 0.04018746 | 0.76951452 | 0.83996624 |
| Feed-Forward NN (Keras) | 0.82740398 | 0.04236546 | 0.79026902 | 0.86453895 |

Table S1: Confidence Interval Scores for AUC of CIP Models on Original Dataset

|  | AUC Mean | AUC Std Dev | AUC 95% CI Lower | AUC 95% CI Upper |
| --- | --- | --- | --- | --- |
| Logistic Regression | 0.8032537 | 0.02946256 | 0.77742863 | 0.82907878 |
| Random Forest | 0.66765807 | 0.02099971 | 0.64925101 | 0.68606513 |
| SVM | 0.77699467 | 0.02016735 | 0.75931722 | 0.79467213 |
| Gradient Boosting | 0.83851711 | 0.03638135 | 0.80662745 | 0.87040677 |
| XGBoost | 0.81204187 | 0.03811615 | 0.77863159 | 0.84545215 |
| LightGBM | 0.80439013 | 0.03948996 | 0.76977565 | 0.8390046 |
| CatBoost | 0.80645731 | 0.03596828 | 0.77492972 | 0.8379849 |
| Feed-Forward NN (Keras) | 0.73059115 | 0.02078955 | 0.71236831 | 0.748814 |

Table S2: Confidence Interval Scores for AUC of CIP Models on Downsampled Dataset

CTX confidence intervals

|  | AUC Mean | AUC Std Dev | AUC 95% CI Lower | AUC 95% CI Upper |
| --- | --- | --- | --- | --- |
| Logistic Regression | 0.62759411 | 0.14042411 | 0.50450695 | 0.75068128 |
| Random Forest | 0.42526088 | 0.19767022 | 0.25199529 | 0.59852647 |
| SVM | 0.64304087 | 0.15338474 | 0.50859322 | 0.77748852 |
| Gradient Boosting | 0.66768162 | 0.14056314 | 0.54447259 | 0.79089064 |
| XGBoost | 0.65958508 | 0.13801604 | 0.53860869 | 0.78056147 |
| LightGBM | 0.70185735 | 0.15111141 | 0.56940236 | 0.83431234 |
| CatBoost | 0.63743291 | 0.17581744 | 0.48332213 | 0.79154369 |
| Feed-Forward NN (Keras) | 0.71887549 | 0.06701449 | 0.6601347 | 0.77761629 |

Table S3: Confidence Interval Scores for AUC of CTX Models on Original Dataset

|  | AUC Mean | AUC Std Dev | AUC 95% CI Lower | AUC 95% CI Upper |
| --- | --- | --- | --- | --- |
| Logistic Regression | 0.64395712 | 0.03850258 | 0.61020812 | 0.67770611 |
| Random Forest | 0.58105913 | 0.05601239 | 0.53196211 | 0.63015615 |
| SVM | 0.62511371 | 0.05076284 | 0.58061812 | 0.6696093 |
| Gradient Boosting | 0.73099415 | 0.06380952 | 0.67506264 | 0.78692566 |
| XGBoost | 0.64918778 | 0.06644172 | 0.59094905 | 0.70742652 |
| LightGBM | 0.58770305 | 0.06147833 | 0.53381492 | 0.64159119 |
| CatBoost | 0.65578298 | 0.07327925 | 0.59155088 | 0.72001507 |
| Feed-Forward NN (Keras) | 0.5662768 | 0.11042005 | 0.46948936 | 0.66306425 |

Table S4: Confidence Interval Scores for AUC of CTX Models on Downsampled Dataset

|  | AUC Mean | AUC Std Dev | AUC 95% CI Lower | AUC 95% CI Upper |
| --- | --- | --- | --- | --- |
| Logistic Regression | 0.48693841 | 0.07843747 | 0.41818494 | 0.55569188 |
| Random Forest | 0.51273418 | 0.06696652 | 0.45403544 | 0.57143293 |
| SVM | 0.53639456 | 0.08818007 | 0.45910132 | 0.61368779 |
| Gradient Boosting | 0.50273928 | 0.09636795 | 0.41826905 | 0.58720951 |
| XGBoost | 0.51277056 | 0.05092016 | 0.46813707 | 0.55740405 |
| LightGBM | 0.49148569 | 0.0480977 | 0.44932619 | 0.53364518 |
| CatBoost | 0.52108844 | 0.05067867 | 0.47666662 | 0.56551025 |
| Feed-Forward NN (Keras) | 0.5098694 | 0.01545825 | 0.49631965 | 0.52341916 |

Table S5: Confidence Interval Scores for AUC of AMP Models on Original Dataset

|  | AUC Mean | AUC Std Dev | AUC 95% CI Lower | AUC 95% CI Upper |
| --- | --- | --- | --- | --- |
| Logistic Regression | 0.59599029 | 0.03328223 | 0.56681713 | 0.62516345 |
| Random Forest | 0.53581302 | 0.02975639 | 0.5097304 | 0.56189564 |
| SVM | 0.54633323 | 0.03145474 | 0.51876194 | 0.57390452 |
| Gradient Boosting | 0.5752295 | 0.04828522 | 0.53290565 | 0.61755336 |
| XGBoost | 0.58596075 | 0.04526407 | 0.54628504 | 0.62563645 |
| LightGBM | 0.56735254 | 0.03235318 | 0.53899373 | 0.59571135 |
| CatBoost | 0.53279519 | 0.03782139 | 0.49964328 | 0.5659471 |
| Feed-Forward NN (Keras) | 0.51595442 | 0.02639598 | 0.49281732 | 0.53909151 |

Table S6: Confidence Interval Scores for AUC of AMP Models on Downsampled Dataset
